# Selective Sweeps in Cattle Genomes in Response to the Influence of Urbanization and Environmental Contamination

**DOI:** 10.3390/genes14112083

**Published:** 2023-11-15

**Authors:** Silpa Mullakkalparambil Velayudhan, Shahin Alam, Tong Yin, Kerstin Brügemann, Andreas Buerkert, Veerasamy Sejian, Raghavendra Bhatta, Eva Schlecht, Sven König

**Affiliations:** 1 Institute of Animal Breeding and Genetics, Justus-Liebig-University Gießen, Ludwigstraße 21 b, 35390 Giessen, Germany; 2 Animal Husbandry in the Tropics and Subtropics, University of Kassel and Georg-August-Universität Göttingen, Steinstr. 19, 37213 Witzenhausen, Germany; 3 Organic Plant Production and Agroecosystems Research in the Tropics and Subtropics, University of Kassel, 37213 Witzenhausen, Germany; 4 National Institute of Animal Nutrition and Physiology (NIANP), Hosur Rd, Chennakeshava Nagar, Adugodi, Bengaluru 560030, India

**Keywords:** adaptation, dairy cattle, environmental contaminants, selection signature, urbanization

## Abstract

A genomic study was conducted to identify the effects of urbanization and environmental contaminants with heavy metals on selection footprints in dairy cattle populations reared in the megacity of Bengaluru, South India. Dairy cattle reared along the rural–urban interface of Bengaluru with/without access to roughage from public lakeshores were selected. The genotyped animals were subjected to the cross-population–extended haplotype homozygosity (XP-EHH) methodology to infer selection sweeps caused by urbanization (rural, mixed, and urban) and environmental contamination with cadmium and lead. We postulated that social-ecological challenges contribute to mechanisms of natural selection. A number of selection sweeps were identified when comparing the genomes of cattle located in rural, mixed, or urban regions. The largest effects were identified on BTA21, displaying pronounced peaks for selection sweeps for all three urbanization levels (urban_vs_rural, urban_vs_mixed and rural_vs_mixed). Selection sweeps are located in chromosomal segments in close proximity to the genes lrand rab interactor 3 (*RIN3*), solute carrier family 24 member 4 (*SLC24A4*), tetraspanin 3 (*TSPAN3*), and proline-serine-threonine phosphatase interacting protein 1 (*PSTPIP1*). Functional enrichment analyses of the selection sweeps for all three comparisons revealed a number of gene ontology (GO) and KEGG terms, which were associated with reproduction, metabolism, and cell signaling-related functional mechanisms. Likewise, a number of the chromosomal segments under selection were observed when creating cattle groups according to cadmium and lead contaminations. Stronger and more intense positive selection sweeps were observed for the cadmium contaminated group, i.e., signals of selection on BTA 16 and BTA19 in close proximity to genes regulating the somatotropic axis (growth factor receptor bound protein 2 (*GRB2*) and cell ion exchange (chloride voltage-gated channel 6 (*CLCN6*)). A few novel, so far uncharacterized genes, mostly with effects on immune physiology, were identified. The lead contaminated group revealed sweeps which were annotated with genes involved in carcass traits (*TNNC2*, *SLC12A5*, and *GABRA4*), milk yield (*HTR1D*, *SLCO3A1*, *TEK*, and *OPCML*), reproduction (*GABRA4*), hypoxia/stress response (*OPRD1* and *KDR*), cell adhesion (*PCDHGC3*), inflammatory response (*ADORA2A*), and immune defense mechanism (*ALCAM*). Thus, the findings from this study provide a deeper insight into the genomic regions under selection under the effects of urbanization and environmental contamination.

## 1. Introduction

Dairy farming plays a major role in satisfying the global demand for high-quality animal protein, especially in developing countries [1]. Most dairy development programs focus on increasing animal productivity via crossbreeding local cattle with high yielding exotic breeds [2]. Though this breeding practice contributes to increased milk production, an unfavorable side effect may be the decline of local cattle breeds that are better adapted to the local environment [2]. Hence, from a breeding perspective, the question arises whether such challenging environmental constraints contribute to natural selection, inducing alterations on the cattle genome. Such effects refer to selection signatures. Selection, natural and artificial, leaves patterns on the genome leading to changes in allele frequencies among populations [3]. Such patterns are termed as “signatures of selection” or “selection footprints”, and can be assessed using varied statistical tests [4]. Genome-wide mapping of selection sweeps in cattle can be broadly categorized into three groups: (a) exploiting high-frequency of derived alleles by Fay and Wu’s *H* Test [5]; (b) assessing population differentiation using diverse methods that consider differences in allele frequency; and (c) tests for long haplotypes applying either EHH [6], iHS [7], or Rsb [8]. The tests based on long haplotypes depict the more recent effects of natural or artificial selection [9].

Urbanization is known as a vital driver of agricultural transition, especially in the Global South, having an impact on the homogeneity of agricultural practices and on intensification or extensification [10]. A few studies evaluated dairy production systems along the rural–urban gradient and phenotypically assessed the effects of urbanization on production, health, and adaptation [10,11,12]. A crucial factor influencing the dairy production system and closely linked to urbanization are environmental contaminants. Urbanization, along with rapid industrial development, implies an accumulation of xenobiotics, especially heavy metals, in the environment [13]. Environmental contamination has a direct deleterious impact on human and animal health, but also indirectly affects human health via animal-based food consumption. Heavy metals like arsenic (As), nickle (Ni), copper (Cu), chromium (Cr), cadmium (Cd), and lead (Pb) are potentially toxic bio-accumulative compounds in dairy production systems [14]. The toxic heavy metals released from industrial waste contaminate the soil, underground water, lakes, and other types of water bodies [14,15]. However, varying levels of heavy metals have been found in the grass growing near these water bodies. Based on an investigation led by [16], it was found that fodder grown near ten major water bodies of urban and peri-urban Bengaluru was contaminated with heavy metals. The concentrations of As, Cd, chromium (Cr), and Pb were reported to be 2.54 ± 1.71 (mean ± standard deviation) mg/kg DM, 0.72 ± 1.79 mg/kg DM, 11.02 ± 15.71 mg/kg DM, and 3.99 ± 5.47 mg/kg DM, respectively. Traces of heavy metals were also reported in four major water bodies of peri-urban Bengaluru by Varalakshmi and Ganeshamurthy [17]. Since fodder was grown on the lakeshores and farmers used this fodder for their animals, the animals could be exposed to heavy metals [16,18,19].

Strong socio-ecological effects such as urbanization and contamination levels might impact animal welfare and animal behavior, causing a production decline that stimulates human intervention towards (counteracting) artificial selection and breeding. Hence, multiple socio-ecological stressors may affect phenotypic and genetic animal traits, not only in a tropical production system context. So far, several studies aimed at understanding physiological (and selection) mechanisms in response to climate change [20]. However, there is a lack of studies evaluating the genetic effects of environmental stressors. Exposure to such diverse stressors might enhance selection towards improved adaptation, indicated through footprints of selection. Nevertheless, developing a relevant research design and identifying a suitable research environment are challenging. To start with, it is imperative to group the animal population to assess the impact of urbanization by, for example, considering the survey stratification index (SSI) developed by Hoffmann et al. [21]. For the assessment of the impact of environmental contamination, it is imperative to determine the concentration of heavy metals in the animals. Considering the practical constraints and ethical concerns in the current study, heavy metal concentration was estimated from hair samples. A multitude of guidelines defines permissible levels for heavy metals in soil, water, fodder, and animal products [22,23,24,25,26]. However, there are no documented thresholds to categorize cadmium and lead concentrations in the hair samples of dairy cows.

The objective of this study was to assess the footprints of selection in dairy cattle reared in the rising megacity of Bengaluru, India, with regard to the effects of urbanization and environmental contamination with the heavy metals Cd and Pb. The chromosomal segments for selective sweeps were annotated with potential candidate genes and physiological pathways representing selection pressures in the context of adaptation and immune response.

## 2. Materials and Methods

### 2.1. Study Location, Sample Collection, and Analysis

The study was conducted in Bengaluru, the capital city of the South Indian state of Karnataka. This region experiences a tropical savanna climate characterized by distinct humid and dry seasons. For the initial phase of the study, a total of 68 farms located along the rural–urban interface of Bengaluru were selected. This interface was represented by two transect lines, a northern and a southern one, which were defined as common space for interdisciplinary research [21]. The rural–urban interface was further distinguished based on the SSI developed by Hoffmann et al. [21] as “urban” (SSI < 0.3), “mixed” (SSI: 0.3–0.5), and “rural” (SSI > 0.5). The dataset comprised of 123, 64, and 53 dairy cows reared in urban, mixed, and rural farms. The selected cows consisted of Holstein Friesian, Jersey, and crossbred cattle (an admixed population of crosses between Holstein Friesian and Jersey with local cattle breeds). The major dietary component of all dairy cows consisted of green forage. The animals were additionally fed with concentrates and with crop residues. Hence, the components of the feeding ration were the same in all herds, but the quality of the green fodder differed, especially due to heavy metal contaminations for the farms with access to public lakeshores. In addition to the criterion of grouping the animals with regard to urbanization (SSI), farm selection considered the access to roughage from public lakeshores for creating cow groups according to the heavy metal contamination of their feed. Hair samples were collected once from the tail of 240 dairy cows reared in the selected farms and stored at room temperature, in clean collection bags, until DNA extraction and heavy metal analyses. 

### 2.2. Heavy Metal Analysis of Hair Samples

Hair samples were collected from the tail of the cow using shears (5 g). Subsequently, the hair samples were transported to the NIANP Animal Nutrition Division laboratory for washing. Immediately after collection, hair samples were washed with tap water until visually clean, and then rinsed with distilled water and then acetone (cleaning agent). Finally, the samples were again thoroughly rinsed with distilled water to attain a high level of purity. Following the cleaning process, the samples were dried in an oven (60 °C, 3 h), and subsequently cut into pieces of 1 cm length and stored at room temperature for heavy metal analysis [27]. 

For the digestion of hairs, the samples underwent microwave- (Anton Paar, Graz, Austria) assisted digestion. Approximately 0.2 g of chopped (1 cm) sample material was placed in a marked polytetrafluoroethylene tube. A volume of 6 mL conc. HNO_3_ (Supra 69%, Roth, Germany) was added, and the vessel was placed in the microwave digester. Then, the digester was pre-heated (100 °C for 10 min, holding time 5 min), heated (180 °C for 10 min, holding time 5 min), digested (190 °C for 5 min, holding time 15 min), and cooled (55 °C for 23 min). After acid digestion, 0.5 mL of HCl (Supra 30%, Roth, Germany) was added to each vessel, along with demineralized water to complete a volume of 25 mL. Subsequently, the digested solid components of the hair samples were recovered by filtration (Whatman paper No. 40) and stored in a polyethylene bottle for heavy metal analysis [28]. A reagent blank sample was also prepared for each batch.

The concentrations of Cd and Pb were determined for hair via inductively coupled plasma–optical emission spectroscopy (ICP-OES) using a Spectrogreen ICP-OES analyzer (Spectro Analytical Instruments GmbH, Kleve, Germany). Argon was used as the plasma gas. Calibration standards were prepared via serial dilution using a dilute HNO_3_ and HCl-matrix-based aqueous solution of 100 mg/L (ppm) (Supelco, Centipur^®^ ICP multi-element standard solution IV, Cat. No. 1.11355.0100, Merck, Germany). Afterwards, from this solution, different concentrations of Cd and Pb (0.005, 0.01, 0.05, 0.1, and 1.0 mg/L) were prepared for the calibration to carry out the hair sample analysis. The analyzed elements were quantified using calibration curves plotted from analytical standards. The limits of detection of the ICP-OES analyzer used in the laboratory were as follows: Cd = 0.000130 mg/L, and Pb = 0.003022 mg/L. Technical replicates were carried out three times for all samples. The analytical method was tested by analyzing the blank samples, and no major interferences were found in the quantitative element analysis.

### 2.3. Genotyping and Quality Control

The DNA was extracted from the hair samples of the 240 dairy cows using the Nucleo-Spin Tissue Kit (Macherey-Nagel GmbH & Co. KG, Düren, Germany) following the manufacturer’s instructions. The samples were genotyped using the *Illumina Bovine 50K SNP BeadChip V2* (96 cows) and the *Illumina Bovine 62K SNP BeadChip* (144 cows). Animals with 50K genotypes were imputed to 62K using Beagle v.5.1 [29]. The PLINK [30] software package was employed to perform the quality control of the genotype data. SNPs located on the sex chromosomes and those with minor allele frequency lower than 0.05 were discarded. Genotyped animals and SNPs with call rates larger than 95% were selected for genomic analysis. The imputed dataset considered 45,054 SNPs from 213 genotyped cows.

### 2.4. Selection Signature Analysis

For the selection signature analysis, group creation was performed according to SSI (rural, mixed, and urban), and according to Cd and Pb concentration in tail hair. Since there are no reports available defining a threshold for heavy metal concentrations in hair samples of dairy cows, the dataset was grouped based on the median values for each heavy metal as determined in the present study (control group: <median (38 animals); treatment group: >median (30 animals)). The grouping for each heavy metal was performed separately, implying that the composition of the cattle groups differed for the Cd and Pb analyses. For the identification of candidate regions under selection, the cross-population–extended haplotype homozygosity (XP-EHH) approach [6] was applied. Selection signature analyses comprised the following group comparisons: urban_vs_rural, urban_vs_mixed, and rural_vs_mixed for the SSI stratification; and Cd-control_vs_ Cd-treatment as well as Pb-control_vs_Pb-treatment for the heavy metals. Using the *rehh* package in R (version 3.1.2; [31,32]), the XP-EHH scores were calculated for each pairwise comparison. The *p*-value for XP-EHH was inferred as a two-sided *p*-value expressed in -log10 scale, wherein values lower than 0.001 (0.1 percentile) were stated to be signatures of selection in a test population [33]. Therefore, by setting the threshold of the top 0.1 percent for both tails of the distribution curve (lower and upper tail), the positive and negative selection signatures were detected. The genes within a window size of 200 kb (100 kb upstream and 100 kb downstream) from the potential regions under selection were annotated using the *Bos taurus* ARSUCD1.2 genome assembly. The functional analyses of the identified genes were accomplished using the default setting in DAVID, and assessing the significantly enriched (*p* < 0.05) GO terms and KEGG pathways. Using the ‘Functional Annotation Clustering’ report in DAVID, similar annotations were grouped together, aiming for a better understanding of the functional pathways.

## 3. Results

### 3.1. Selection Signatures According to SSI Grouping

**Figure 1:**
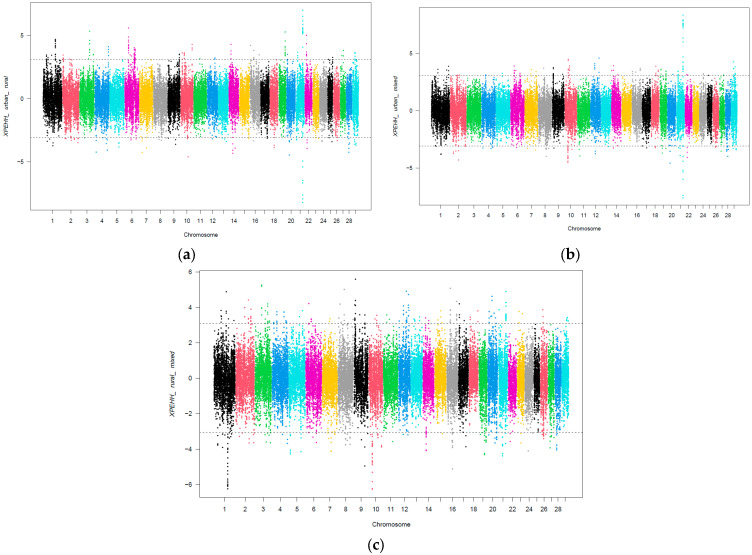
Distribution of XP-EHH values across the genome for urban_vs_rural (**a**), urban_vs_mixed (**b**), and rural_vs_mixed (**c**) SSI comparison groups. The *x*-axis depicts the SNP position in the genome, and the *y*-axis depicts the XP-EHH values. The dotted lines indicate the top 0.1 percentile for positive and bottom 0.1 percentile for negative selection for each comparison. SNPs over these dotted lines indicate significant selection sweeps. Colors demarcate different chromosomes and have no particular significance.

A number of selection sweeps were identified when comparing the genomes of cattle located in rural, mixed, or urban regions (Figure 1).

The selection sweeps in cows reared in urban regions are represented by negative XP-EHH values for the urban_vs_rural (Figure 1a) and urban_vs_mixed (Figure 1b) comparisons. The positive XP-EHH values for the urban_vs_mixed and rural_vs_mixed comparisons represent the selection sweeps for cows in mixed regions. Lastly, the selection signatures for rural animals are represented by the positive XP-EHH values for the urban_vs_rural comparison and negative XP-EHH values for the rural_vs_mixed comparison. Upon assessing the XP-EHH scores and the threshold set (top 0.1 percentile of positive and bottom 0.1 percentile of negative values, respectively), 165, 125, and 186 genomic regions were observed to be positively selected for the comparisons urban_vs_rural, urban_vs_mixed, and rural_vs_mixed, respectively. Likewise, 90, 85, and 187 genomic regions were found to be negatively selected for the urban_vs_rural, urban_vs_mixed, and rural_vs_mixed comparisons, respectively. The gene annotations revealed 119, 97, and 198 genes to be under positive selection for the urban_vs_rural, urban_vs_mixed, and rural_vs_mixed comparisons, respectively. Likewise, 135, 155, and 235 genes were observed to be associated with negative selection sweeps in dairy cattle for the urban_vs_rural, urban_vs_mixed, and rural_vs_mixed comparisons, respectively. BTA21 was the chromosome displaying the most obvious selection signals for all three comparisons. Consequently, we strongly focused on the gene annotations of the respective chromosomal segments. The gene annotations for the selection sweeps on BTA21 for urban_vs_rural, urban_vs_mixed, and rural_vs_mixed are summarized in Table 1. The most interesting genes with regard to mechanisms of adaptation, resistance, and resilience were Ras and Rab interactor 3 (*RIN3*), Solute carrier family 24 member 4 (*SLC24A4*), Tetraspanin 3 (*TSPAN3*), and Proline-serine-threonine phosphatase interacting protein 1 (*PSTPIP1*). 

**Figure 2:**
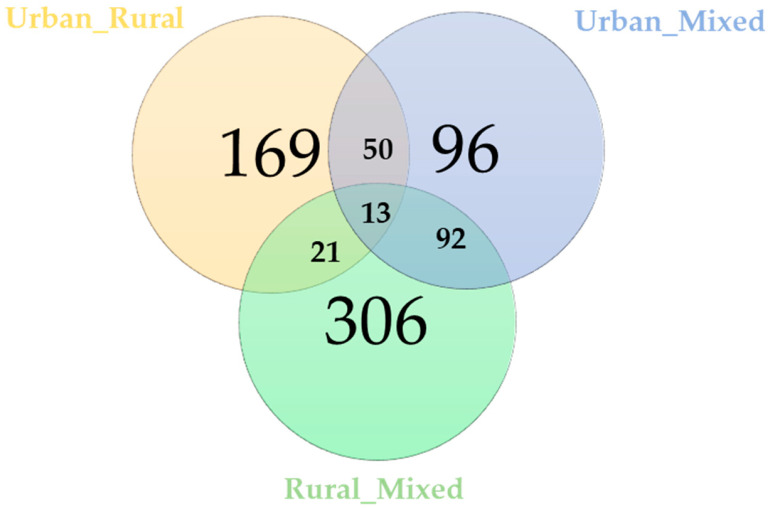
Venn diagram depicting the genes common and unique to the urban_vs_rural, urban_vs_mixed, and rural_vs_mixed comparisons.

Clustering the genes associated with the positive and negative selection sweeps for each comparison, 13 genes were identified to be common for the urban_vs_rural, urban_vs_mixed, and rural_vs_mixed comparisons (Figure 2; Supplementary Table S1). On the other hand, 169, 96, and 306 genes were unique for each comparison, i.e., urban_vs_rural, urban_vs_mixed, and rural_vs_mixed, respectively (Figure 2).

### 3.2. Functional Analysis of Urbanization Effects

Supplementary Figure S1 depicts the GO terms associated with positive and negative selection sweeps for each of the group comparisons. None of the KEGG pathways were observed to be significantly enriched when assessing the positive selection sweeps for urban_vs_rural, urban_vs_mixed, and rural_vs_mixed comparisons. A number of significantly enriched KEGG pathways were observed for the negative selection sweeps for urban_vs_rural, urban_vs_mixed, and rural_vs_mixed comparisons. The functional annotation clustering for the urban_vs_rural negative selection sweep comparison revealed a number of KEGG pathways as depicted in Table 2, with an enrichment score of 1.67. The KEGG pathways *ovarian steroidogenesis* (bta04913) and *cortisol synthesis and secretion* (bta04927) were significantly enriched (enrichment score: 0.95) for the urban_vs_mixed negative selection sweep. Likewise, *cushing syndrome* (bta04934), *cortisol synthesis and secretion* (bta04927), *prolactin signaling pathway* (bta04917), and *ovarian steroidogenesis* (bta04913) were the significantly enriched KEGG pathways for the rural_vs_mixed negative selection.

### 3.3. Selection Signatures According to Heavy Metal Contamination Grouping

**Figure 3:**
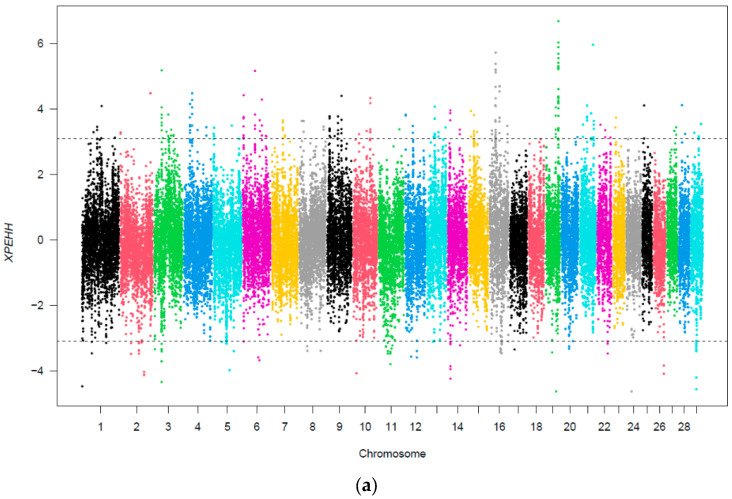

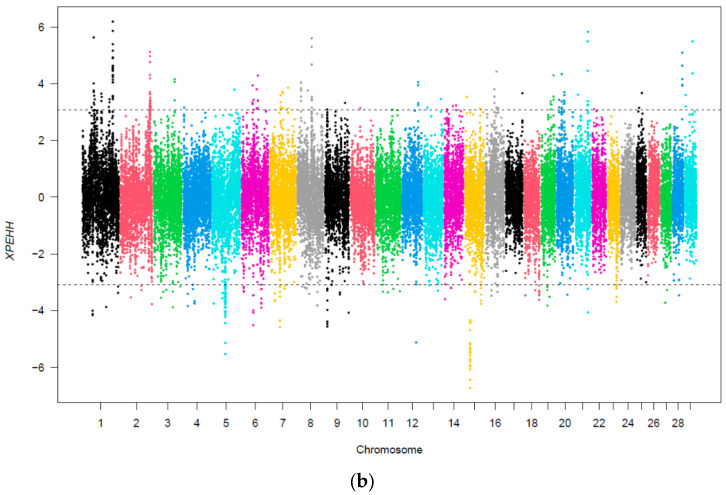
Distribution of XP-EHH values across the genome for the cadmium (**a**) and lead (**b**) comparison groups. The *x*-axis depicts the SNP position in the genome, and the *y*-axis depicts the XP-EHH values. The dotted lines indicate the top 0.1 percentile for positive and bottom 0.1 percentile for negative selection for each comparison. SNPs over these dotted lines indicate significant selection sweeps. Colors demarcate different chromosomes and have no particular significance.

To assess the signatures of selection in response to heavy metal contamination, the dataset was grouped based on the median value for each heavy metal. The two heavy metals considered in the present study, cadmium and lead, were significantly correlated with a coefficient of 0.30 (*p* = 0.004). The average cadmium and lead concentrations for the current dataset were 0.12 mg/L and 2.10 mg/L, respectively, indicating a significant difference (*p* < 0.01). Furthermore, the cadmium and lead concentrations between the case and the respective control groups differed significantly (*p* < 0.001). Using the XP-EHH methodology, a number of selection sweeps were identified when creating cattle groups according to cadmium and lead contaminations (Figure 3).

The analysis revealed 221 and 169 genomic regions under positive selection for Cd and Pb group comparisons, respectively. Further gene annotation of these regions revealed 207 potential candidate genes for the cadmium grouping, and 161 potential candidate genes for the lead grouping. 

The GO terms associated with both heavy metal comparisons were broadly associated with cell signaling and functioning mechanisms (Table 3). 

ATPase activity (GO: 0016887; MF), microtubule motor activity (GO: 0003777; MF), and kinesin complex (GO: 0005871; CC) were the significantly enriched gene ontology terms with regard to the cadmium comparison. These terms contained the following genes: DEAD-box helicase 24 (*DDX24*), RecQ like helicase 5 (*RECQL5*), Kinesin family member 18B (*KIF18B*), Midasin AAA ATPase 1 (*MDN1*), RNA sensor RIG-I (*RIGI*), Kinesin family member 5B (*KIF5B*), and Kinesin family member 12 (*KIF12*). Similarly, the genes associated with the GO terms for lead included troponin C2 fast skeletal type (*TNNC2*), opioid receptor delta 1 (*OPRD1*), solute carrier family 12 member 5 (*SLC12A5*), γ-aminobutyric acid type A receptor subunit alpha4 (*GABRA4*), 5-hydroxytryptamine receptor 1D (*HTR1D*), protocadherin β-4-like (*LOC104968820*), solute carrier organic anion transporter family member 3A1 (*SLCO3A1*), TEK receptor tyrosine kinase (*TEK*), kinase insert domain receptor (*KDR*), protocadherin γ subfamily C, 3 (*PCDHGC3*), adenosine A2a receptor (*ADORA2A*), opioid binding protein/cell adhesion molecule like(*OPCML*), *SLC24A4*, and activated leukocyte cell adhesion molecule (*ALCAM*). No KEGG terms were observed to be significantly enriched for any of the heavy metal comparisons.

## 4. Discussion

### 4.1. Selection Sweeps Due to Urbanization

The demand for animal-based food is substantially increasing in developing countries given the rapid population growth, increased income, and increasing urbanization [34,35]. Most of the genetic research conducted in dairy cattle populations in the tropics focused on production traits in response to farm management, reproduction, climate change, and disease prevalence. Only a few studies focused on the challenging environmental effects that urbanization and feed contaminants have on dairy production, by ignoring genomic mechanisms. Some of these assessed phenotypic trait association analyses and considered stratification according to herd productivity [11], feed efficiency [10], cattle health [11], heat stress response [20], and milk quality [16] along the rural–urban transects of Bengaluru. However, to the best of our knowledge, this is the first study assessing the signatures of selection in dairy cattle reared along the rural–urban interface and exposed to environmental contamination. This study is therefore providing relevant insights into the genomic mechanisms of adaptation that are (indirectly) fostered when dairy animals are subjected to environmental constraints over prolonged periods of time.

Our study revealed significant signatures of selection when comparing cattle reared in urban, rural, and mixed regions. Strong selection signals and a high density of selection sweeps were observed on BTA 21, especially in chromosomal segments located in close proximity to genes with varied biological functions. Among these genes, *SLC24A4* and *CPSF2* are potential candidate genes reflecting selection due to urbanization. Significant SNPs associated with these genes were detected for both the urban_vs_rural and urban_vs_mixed comparisons. *SLC24A4* encodes a member of potassium-dependent sodium or calcium exchanger protein family and was associated with hair color and pigmentation-related traits [36]. In genomic studies, specific variants of *SLC24A4* were significantly associated with milk urea concentration [37], body conformation [38], reproduction [39], and production [40] traits in cattle. Likewise, gene *CPSF2*, with effects on the RNA binding activity, was significantly associated with lipid metabolism in cattle [41]. When assessing the selection sweeps and annotated genes for group comparisons including the mixed region, *SLC24A4* and *RIN3* were shared genes for both the urban_vs_mixed and rural_vs_mixed comparisons. *RIN3* is a Ras interaction-interference effector protein, which plays a crucial role in regulating bone metabolism in mice [42]. In cattle, *RIN3* was associated with growth traits [43], and reported to have a pleotropic effect on growth- and conformation-related traits (weaning weight, fatness, and conformation) [44]. Likewise, *SLC24A4* and *RIN3* were also potential candidate genes for the rural cattle population. 

The functional analysis of the annotated genes revealed a diverse range of gene ontology terms and KEGG pathways for each SSI comparison. The pathways were linked with reproduction, metabolism, and cell signaling-associated functional mechanisms. Such physiological mechanisms might explain the observed phenotypic differences in cattle along the SSI in Bengaluru for productive performances and hygiene traits [11,12]. It is interesting to note that similar pathways are enriched in urban and rural cows, when compared to the population from the mixed region (urban_vs_mixed and rural_vs_mixed; Table 2). *Cortisol synthesis and secretion* (bta04927), *prolactin signaling pathway* (bta04917) and *ovarian steroidogenesis* (bta04913) were among these pathways. All these pathways included genes associated with steroid and other reproductive hormones (cytochrome P450 family 17 subfamily A member 1 (*CYP17A1*), glycogen synthase kinase 3 β (*GSK3B*), adenylate cyclase 2 (*ADCY2*), and Wnt family member 9B (*WNT9B*)). Additionally, the analysis suggested the potential candidate gene SHC adaptor protein 3 (*SHC3*), which was associated with residual feed intake in beef cattle [45]. Furthermore, variants of *SHC3* enhanced the adaptability of thermo-tolerant cattle to maintain production and reproduction in harsh tropical environments [46].

### 4.2. Selection Sweeps Due to Heavy Metal Contamination

Another consequence of rapid urbanization is the rise in heavy metal pollution through industries [14,47]. Garment factories, electroplating, industries, and distilleries are some of the small-scale water-polluting industries in Bengaluru [48]. These industries contaminate groundwater and nearby water bodies with heavy metals [14,49,50]. Due to the increasing importance of animal welfare and health, several studies addressed the challenge of heavy metal accumulation in animals, livestock feed, and livestock products [14,51]. However, there are scanty reports evaluating this impact from a genetic perspective [52,53].

Our analysis revealed 221 and 169 genomic regions under strong and/or recent positive selection in response to Cd and/or Pb contamination. These potential regions were linked to 207 and 161 genes, respectively. Stronger and intense positive selection sweeps were observed for Cd on BTA 16 and BTA19. The respective chromosomal segments harbor genes significantly influencing the somatotropic axis (growth factor receptor-bound protein 2 (*GRB2*)) and cell ion exchange (chloride voltage-gated channel 6 (*CLCN6*)). Previous studies associated variants of these genes with candidate regions representing selection processes in cattle [54,55]. It was interesting to observe a few uncharacterized genes in this region (ENSBTAG00000052901, ENSBTAG00000050070, and ENSBTAG00000030910), which were grouped as membrane co-factor proteins and involved in immune response mechanisms. Effects on immunity might be a novel finding obtained from the present study, encouraging further evaluations and validations in this regard. 

The functional analyses of the annotated genes based on Cd contamination grouping revealed significant enrichment of the GO terms ATPase activity (GO: 0016887; MF), microtubule motor activity (GO: 0003777; MF), and kinesin complex (GO: 0005871; CC). Genes associated with these terms included *DDX24*, *RECQL5*, *KIF18B*, *MDN1*, *RIGI*, *KIF5B*, and *KIF12*. *DDX24* belongs to the DEAD box containing RNA helicases [56]. Based on their distribution patterns, some genes belonging to this family were associated with embryogenesis, spermatogenesis, and cellular growth and division [57]. Furthermore, *DDX24* was a regulator of p53 transcriptional activity [56]. The p53 transcriptional regulator plays a crucial role in adapting gene expression programs to maintain cellular homeostasis and genome integrity during stress [58]. Therefore, the identification of this gene through the current selection signature analysis provides a vital insight towards the selection of animals with adaptation to environmental contaminants that act as a stressor. Similarly, variants of *RECQL5* are associated with fertility in dairy cows [59], while *KIF18B* contributes to milk lactose alterations in dairy cows [60].

The GO analyses for annotated genes based on the selection sweeps according to Pb contamination grouping were associated with cell adhesion and calcium ion-binding processes. The detected genes were associated with carcass traits (*TNNC2*, [61]; *SLC12A5*, [62]; and *GABRA4*, [63]), milk traits (*HTR1D*, [64]; *SLCO3A1*, [65]; *TEK*, [66]; and *OPCML*, [67]), reproduction (*GABRA4*, [68]), hypoxia/stress response (*OPRD1*, [69]; and *KDR*, [70]), cell adhesion (*PCDHGC3*, [71]), inflammatory response (*ADORA2A*, [72]), and immune defense mechanism (*ALCAM*, [73]). The links between selection signals due to environmental contamination and signals for disease resistance and immunity suggest further studies in this regard, namely, detailed genome comparisons that consider environmental stressors. The challenging production context of a rapidly growing tropical megacity with a pronounced social-ecological gradient might be the ideal “research lab” in this regard.

## 5. Conclusions

To our knowledge, this was a first study aiming at the identification of the genomic selection footprints of dairy cows in response to urbanization and environmental contaminants. The genomic regions under selection due to urbanization are involved in reproduction, metabolism, and cell signaling and functional mechanisms. In the context of adaption, previously described genes including *GRB2* and *CLCN6* could be verified based on the cadmium contamination grouping. Furthermore, this grouping contributed to the detection of novel and uncharacterized genes including ENSBTAG00000052901, ENSBTAG00000050070, and ENSBTAG00000030910, which influence the somatotropic axis, cell ion exchange, and immune response. Selection sweeps according to lead contamination grouping also indicated genomic regions affecting phenotypes of carcass traits, reproduction, hypoxia/stress response, cell adhesion, inflammatory response, and immune defense. Despite the quite small sample size used, the present study demonstrates a novel approach to infer genomic mechanisms of adaptation and genomic responses to environmental challenges.

## Figures and Tables

**Table 1:** Overview of genes associated with selection sweeps on BTA21.

Comparison	Gene Details
Urban_vs_Rural Positive (top 0.1 percentile)	Solute carrier organic anion transporter family member 3A1 (*SLCO3A1*) Ras and Rab interactor 3 (*RIN3*) Solute carrier family 24 member 4 (*SLC24A4*)
Urban_vs_Rural Negative (bottom 0.1 percentile)	Cleavage and polyadenylation specific factor 2 (*CPSF2*) Solute carrier family 24 member 4 (*SLC24A4*) ADAM metallopeptidase with thrombospondin type 1 motif 17 (*ADAMTS17*)
Urban_vs_Mixed Positive (top 0.1 percentile)	Solute carrier organic anion transporter family member 3A1 (*SLCO3A1*) Solute carrier family 24 member 4 (*SLC24A4*) Ras and Rab interactor 3 (*RIN3*) Family with sequence similarity 174 member B (*FAM174B*) Chromodomain helicase DNA binding protein 2 (*CHD2*)
Urban_vs_Mixed Negative (bottom 0.1 percentile)	DET1 partner of COP1 E3 ubiquitin ligase (*DET1*) Tetraspanin 3 (*TSPAN3*) DEAD-box helicase 24 (*DDX24*) Pseudopodium enriched atypical kinase 1 (*PEAK1*) Solute carrier family 24 member 4 (*SLC24A4*) Ankyrin repeat and SOCS box containing 2 (*ASB2*) Cleavage and polyadenylation specific factor 2 (*CPSF2*) OTU deubiquitinase, ubiquitin aldehyde binding 2 (*OTUB2*) Reticulocalbin 2 (*RCN2*) Coiled-coil domain containing 197 (*CCDC197*) Mitochondrial ribosomal protein L46 (*MRPL46*) Mitochondrial ribosomal protein S11 (*MRPS11*) Proline-serine-threonine phosphatase interacting protein 1 (*PSTPIP1*) Family with sequence similarity 181 member A (*FAM181A*)
Rural_vs_Mixed Positive (top 0.1 percentile)	Solute carrier family 24 member 4 (*SLC24A4*) Ceramide synthase 3 (*CERS3*) Ras and Rab interactor 3 (*RIN3*) Ankyrin repeat and SOCS box containing 7 (*ASB7*) Lines homolog 1 (*LINS1*)
Rural_vs_Mixed Negative (bottom 0.1 percentile)	Tetraspanin 3 (*TSPAN3*) Pre-mRNA processing factor 39 (*PRPF39*) FKBP prolyl isomerase 3 (*FKBP3*) FA complementation group M (*FANCM*) MIS18 binding protein 1 (*MIS18BP1*) Pseudopodium enriched atypical kinase 1 (*PEAK1*) Reticulocalbin 2 (*RCN2*) Proline-serine-threonine phosphatase interacting protein 1 (*PSTPIP1*) TOG array regulator of axonemal microtubules 1 (*TOGARAM1*)

**Table 2:** The Kyoto Encyclopedia of Genes and Genomes (KEGG) pathways obtained based on functional annotation clustering using DAVID for the negative selection sweeps of urban_vs_rural, urban_vs_mixed, and rural_vs_mixed group comparisons.

KEGG Term	Gene Count	Raw *p*-Value	Fold Enrichment
**Urban_vs_Rural (Enrichment Score: 1.67)**			
bta00480: Glutathione metabolism	4	0.01	10.92
bta00982: Drug metabolism—cytochrome P450	4	0.01	10.92
bta05204: Chemical carcinogenesis—DNA adducts	4	0.01	10.41
bta00980: Metabolism of xenobiotics by cytochrome P450	4	0.01	10.10
bta01524: Platinum drug resistance	4	0.01	8.68
bta00983: Drug metabolism—other enzymes	5	9.54 × 10^−4^	11.13
bta05418: Fluid shear stress and atherosclerosis	5	0.01	5.83
bta05207: Chemical carcinogenesis—receptor activation	5	0.04	3.92
bta05208: Chemical carcinogenesis—reactive oxygen species	5	0.05	3.53
**Urban_vs_Mixed (Enrichment Score: 0.95)**			
bta04913: Ovarian steroidogenesis	3	0.04	8.69
bta04927: Cortisol synthesis and secretion	3	0.05	8.17
**Rural_vs_Mixed (Enrichment Score: 1.96)**			
bta04934: Cushing syndrome	8	0.00	4.67
bta04927: Cortisol synthesis and secretion	5	0.01	6.88
bta04917: Prolactin signaling pathway	5	0.01	5.48
bta04913: Ovarian steroidogenesis	4	0.03	5.85

**Table 3:** Gene ontology (GO) terms obtained based on functional annotation clustering using DAVID for cadmium and lead group comparisons.

GO Category	GO Term	Gene Count	Raw *p*-Value	Fold Enrichment
**Cadmium (control_vs_treatment)**				
Molecular Function	GO:0016887: ATPase activity	7	0.03	3.02
	GO:0003777: Microtubule motor activity	3	0.04	8.84
Cellular Component	GO:0005871: Kinesin complex	3	0.04	9.14
**Lead (control_vs_treatment)**				
Biological Process	GO:0007156: Homophilic cell adhesion via plasma membrane adhesion molecules	11	3.27 × 10^−8^	11.68
	GO:0007155: Cell adhesion	11	1.18 × 10^−4^	4.69
Molecular Function	GO:0005509: Calcium ion binding	12	0.00	2.82
Cellular Component	GO:0005887: Integral component of plasma membrane	19	3.53 × 10^−5^	3.11

## Data Availability

The anonymized data set that forms the basis of this article is available through the institutional repository at the University of Göttingen. For scientific purposes, access will be provided upon written request to the corresponding author.

## Supplementary Materials

The following supporting information can be downloaded at: https://www.mdpi.com/article/10.3390/genes14112083/s1, Figure S1: Functional annotation clustering of the gene ontology terms (BP: Biological process, CC: Cellular Component, and MF: Molecular Functions) for the selection sweeps for urban_vs_rural (positive (a), negative (b)), urban_vs_mixed (positive (c), negative (d)), and rural_vs_mixed (positive (e), negative (f)) comparisons; Table S1: Details of genes that were common for urban_rural, urban_mixed, and rural_mixed selection signature comparisons.
