# Supplementary material for: Selective Sweeps in Cattle Genomes in Response to the Influence of Urbanization and Environmental Contamination
Source: Genes (Basel). 2023 Nov 15;14(11):2083. doi: 10.3390/genes14112083 (PMC10671461; doi:10.3390/genes14112083)
Supplement: Supplementary file 1 [file genes-14-02083-s001.zip › genes-2704487-supplementary.pdf]

Supplementary Materials:

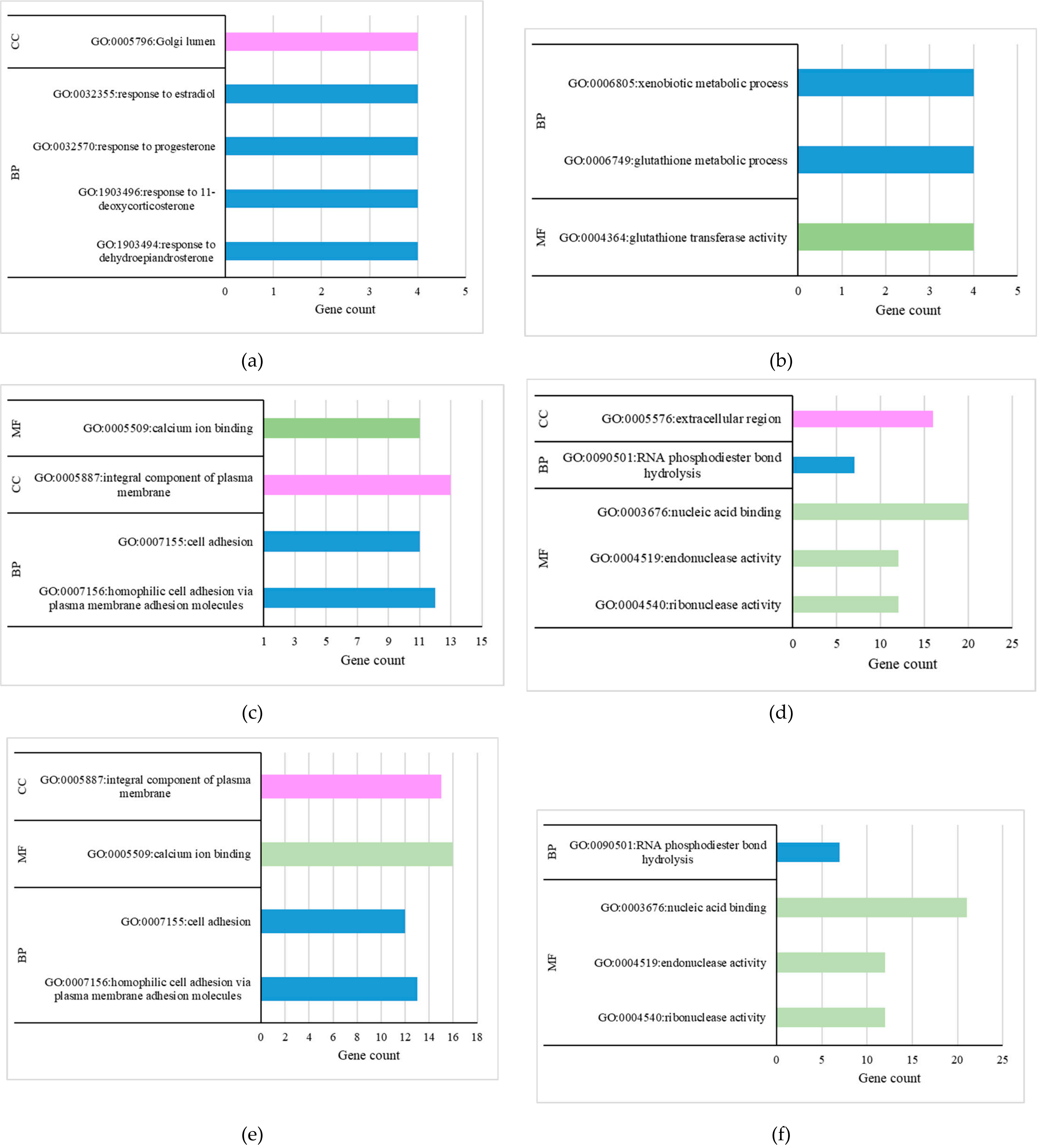

Supplementary Figure S1. Functional annotation clustering of the gene ontology terms (BP: Biological process, CC: Cellular Component and MF: Molecular Functions) for the selection sweeps for urban_vs_rural (positive (a), negative (b)), urban_vs_mixed (positive (c), negative (d)) and rural_vs_mixed (positive (e), negative (f)) comparisons.

Supplementary Table S1: Details of genes that were common for urban_rural, urban_mixed and rural_mixed selection signature comparisons

**Ensemble Gene ID**	**Gene Name**
ENSBTAG00000006620	solute carrier family 24 member 4(*SLC24A4*)
ENSBTAG00000010416	Ras and Rab interactor 3(*RIN3*)
ENSBTAG00000010758	small integral membrane protein 5(*SMIM5*)
ENSBTAG00000011713	Myosin XVB (*A0A3Q1M5U4_BOVIN*)
ENSBTAG00000011715	RecQ like helicase 5(*RECQL5*)
ENSBTAG00000011717	SAP30 binding protein(*SAP30BP*)
ENSBTAG00000014248	MIER family member 3(*MIER3*)
ENSBTAG00000014964	galactokinase 1(*GALK1*)
ENSBTAG00000018169	integrin subunit beta 4(*ITGB4*)
ENSBTAG00000020067	LLGL scribble cell polarity complex component 2(*LLGL2*)
ENSBTAG00000024909	H3.3 histone B(*H3-3B*)
ENSBTAG00000047510	small integral membrane protein 6(*SMIM6*)
ENSBTAG00000051466	unc-51 like kinase 4(*ULK4*)
